# Enhancing Alzheimer's Care at Christopher House Through Sensory Stimulation Therapy

**DOI:** 10.1002/alz70858_104089

**Published:** 2025-12-26

**Authors:** Jackson Nguyen, Azizah Ramsingh, Sean Feeny, Tsitsi Masvawure

**Affiliations:** ^1^ Worcester Polytechnic Institute, Worcester, MA, USA

## Abstract

**Background:**

Sensory stimulation has emerged as a promising nonpharmacological intervention in geriatric care, particularly for individuals with Alzheimer's disease (AD). Sensory impairments in hearing, smell, and touch are common in AD and contribute to behavioral challenges, mood disturbances, and cognitive decline. Thus, engaging in meaningful and purposeful activities to stimulate these senses is essential for their long‐term functionality. However, many nursing centers, including Christopher House in Massachusetts, lack access to such interventions, leaving residents—especially those in advanced stages of AD—at risk for sensory deprivation. This 6‐month research project aims to design a personalized sensory stimulation program tailored to the Brookside dementia unit at Christopher House, utilizing a Snoezelen multisensory environment. This intervention incorporates soft lighting, soothing sounds, calming scents, and tactile textures to engage residents meaningfully.

**Method:**

Our methodology includes a systematic review of 20 peer‐reviewed Snoezelen studies, encompassing randomized controlled trials (RCTs), quasi‐experimental designs, and ethnographic analyses between 2004 and 2024. Additionally, we conducted virtual interviews with 16 leading experts in sensory research and Alzheimer's care worldwide to gather diverse perspectives and insights.

**Result:**

Through inductive thematic analysis of these findings, we will develop an evidence‐based, actionable recommendations in the form of a personalized multisensory program.

**Conclusion:**

While designed for Christopher House, this adaptable framework offers potential for implementation in other Alzheimer's care facilities, presenting a scalable solution to enhance the quality of life for individuals with AD.

